# What Effect Do Pulmonary Micronodules Detected at Presentation in Patients with Osteosarcoma Have on 5-Year Overall Survival?

**DOI:** 10.3390/jcm10061213

**Published:** 2021-03-15

**Authors:** Reid Davison, Fadi Hamati, Paul Kent

**Affiliations:** 1 Rush University Medical College, Chicago, IL 60612, USA; fadi_Hamati@rush.edu (F.H.); paul_kent@rush.edu (P.K.); 2 Rush Medical Center, Pediatric Hematology/Oncology, Rush University Medical Center, Chicago, IL 60612, USA

**Keywords:** osteosarcoma, sarcoma, neoplasm metastasis, solitary pulmonary nodule, survival

## Abstract

For osteosarcoma, staging criteria, prognosis estimates, and surgical recommendations have not yet changed to reflect increasingly sensitive computed tomography (CT) imaging. However, the frequent identification of micronodules (<5 mm) on presentation leaves clinicians in a difficult position regarding the need to biopsy, resect, or follow the lesions and whether to consider the patient metastatic or non-metastatic. Our objective was to compare the 5-year overall survival rates of patients with osteosarcoma with non-surgically resected lung micronodules on presentation to patients without micronodules to guide community oncologists faced with this common dilemma. We collected data retrospectively on all newly diagnosed osteosarcoma patients, aged less than 50, treated at Rush University Hospital over 25 years without pulmonary nodules >10 mm or pulmonary surgical intervention. Kaplan–Meier curves showed there was no difference in 5-year overall survival in patients with any size nodule <5 mm compared to patients with no nodules. Additionally, our study showed a survival advantage for those who presented with 0 or 1 nodule (90%) compared to ≥2 nodules (53%). Our data suggest surgery may not be necessary for singular nodules <5 mm identified on presentation, and that these patients behave more like “localized” patients than metastatic patients.

## 1. Introduction

Osteosarcoma (OST) is one of the most common non-hematopoietic bone sarcomas with an annual incidence of 800 to 900 new cases in the United States, with about half of these cases involving children and teens; however, people of any age can be affected [1,2,3,4]. The 5-year overall survival rate of patients with OST ranges from 28% to 70% [5,6]. This variation has been attributed to several factors including metastasis at diagnosis, age, tumor location, tumor size, and percent necrosis at primary surgery. The biggest improvements are from adjuvant chemotherapy for OST patients classified as “localized” at diagnosis. Because the lungs are the most common site for metastases, all patients with biopsy positive OST receive an initial lung computed tomography (CT) scan. However, it has not been well established how the discovery of lung micronodules at the time of diagnosis effects the 5-year overall survival or the management of patients with OST, since the dichotomy between “localized” and “metastatic” has become blurred. Since 1996 the Children’s Oncology Group (COG) and the National Comprehensive Cancer Network (NCCN) have had an inconsistent and invalidated definition of “pulmonary metastasis” for subcentimeter lesions based on initial CT imaging for OST [7]. In patients newly diagnosed with OST, the dichotomous classification of the disease as “localized” or “metastatic” has implications with respect to surgical intervention, clinical trial participation, and presumed prognosis.

The widespread adoption of thin-slice CT scans in the last 25 years has revealed pulmonary micronodules (defined as nodules < 10 mm) not previously appreciated [8]. Fishbach et al. demonstrated that there is approximately double the detection rate of 2–5 mm nodules with 1.25 mm CT slices compared to 5 mm CT slices [8]. These authors make the point that although thin-slice CT will raise the sensitivity for small lung nodule detection, it “remains problematic about how the detection of small nodules will affect patient outcome” [8]. This category of nodules is important as many consider lung nodules <10 mm as ‘non-measurable’ and cannot be used as ‘target lesions’ by Response Evaluation Criteria In Solid Tumors (RECIST 1.1) criteria [9]. These lesions may increase, decrease, or disappear without resolving the question of their etiology, which may include transient atelectasis, recent infection, arteriovenous malformations (AVM), granulomatosis, or simply different degrees of sensitivity by the CT scanner or the radiologist [9,10], in addition to metastatic disease. However, the frequent identification of micronodules on presentation leaves practicing community clinicians a difficult position regarding the need to biopsy, resect, or follow the lesions, and whether to consider the patient metastatic or non-metastatic for purposes of study enrollment and prognosis counselling [9]. Osteosarcoma was selected as our population of choice because surgery is often recommended as a curative intervention for detectable lung lesions. In fact, open bilateral surgical thoracic exploration is still recommended in many protocols. The assumed, but unproven assertion is that surgical resection of tiny lung nodules benefits the patient.

Staging criteria, prognosis estimates, and surgical recommendations have not yet changed to reflect the increase in incidence of detecting lung nodules. The “New Response Evaluation Criteria in Solid Tumors: Revised RECIST guideline (version 1.1)” considers lung nodules < 10 mm as ‘non-measurable,’ and many clinical trials require RECIST criteria, i.e., lesions > 10 mm for enrollment [9]. At the COG Annual Meeting in 2008, the COG identified “small pulmonary nodules” as an area requiring additional research; to date, however, the literature remains scant. Additionally, the relationship between percent necrosis of the primary tumor after 2 cycles of chemotherapy, which is an important prognostic indicator for patients with OST, [11,12,13,14] and lung nodule management has yet to be examined.

The few studies that address this issue do not include all patients under the age of 50 years, do not limit it to initial CT findings (which is important because development of new lesions after initial presentation is associated with progressive, chemotherapy resistant disease), do not limit it to OST (an important consideration since surgery is seen as necessary for curative intent in OST, unlike many other sarcomas), nor have they studied the effect of surgery versus observation of micronodules on overall survival [15,16]. For example, Kusam et al., retrospectively studied 27 OST and 8 Ewings sarcoma patients, less than 25 years of age, who had biopsies of lung nodules presenting at any time, and found that nodules >5 mm predicted malignancy, and up to 1/3 of those <5 mm when found in connection to large nodules (>5 mm) were malignant but only two patients with only <5 mm nodules found to be malignant after biopsy [17]. We do not know if these micronodules were present at diagnosis, therefore affecting management, or appeared later, nor if the surgery effected survival. Ghosh, et al., retrospectively studied 104 mostly adult OST patients with “indeterminate nodules” (defined as radiologist unable to call them “benign,” or “metastatic,” and non-calcified <10 mm), found survival for “indeterminate” nodules similar to those classified as “without metastasis,” [18] but we do not know who had surgery and which micronodules were not counted because they were initially read as “benign” or “malignant” or “calcified” nor if size, location, and number of nodules was analyzed.

We sought to address some of these shortcomings using our own extensive database. Our institution has maintained a combined adult and pediatric oncology sarcoma program for 40 years, with a longstanding combined weekly sarcoma tumor board, a standard of practice of treating all OST patients under 50 uniformly according to standard COG (or current pediatric) protocols that have not changed for the last 25 years.

## 2. Materials and Methods

Under an Institutional Review Board (IRB) approved protocol we retrospectively collected data on all newly diagnosed OST patients, aged ≤50, treated at Rush University Hospital over 25 years from 1995–2020 who had an initial CT chest report available for review within two months of diagnosis. Subjects were excluded if they had a history of other prior cancer treatment, any lung nodule >10 mm, or other metastatic disease. Diagnosis date was defined as the date of biopsy-proven OST. No patients were excluded for being lost to follow up. All patients underwent similar treatment following COG protocols, which includes surgical excision of the primary tumor with neoadjuvant (induction chemotherapy with 2 cycles of cisplatin, doxorubicin, and high dose methotrexate (MAP)) and adjuvant (4 cycles of MAP) chemotherapy.

Patient demographics (sex, age at diagnosis, ethnicity, primary tumor location, type of primary resection surgery, percent necrosis, and dates last known alive and dates of death) were collected by reviewing medical records. Initial CT scan reports within the first month of diagnosis were identified and reviewed for number of nodules, size of the nodules, location, and laterality. Micronodule size was defined by radiologist CT report. If size of nodule was not listed, authors reviewed CT to determine size. “Good Responders” and “Poor Responders” were defined as in the COG protocols and the Huvos scoring system as ≥90% or <90% necrosis at definitive surgery.

Over the 25-year inclusion period, corresponding to the era of consistent thin-slide CT scanning at our institution, patients who fit these criteria were divided into cohorts based on the number of nodules on the initial CT scan, the largest nodules on presentation, and the number of lobes involved. In addition, an “additive micronodule cohorts,” based on RECIST 1.1 for Solid tumors [9] “sum of target lesions at baseline” paradigm, were constructed, where, for example, a patient with 3 mm and 4 mm nodules would be represented as 7 mm. Kaplan–Meier survival curves were constructed and compared between the cohorts. Data was analyzed using R-Studio version (RStudio: Integrated Development for R. RStudio, PBC, Boston, MA, USA), and an alpha value of 0.05 was considered significant. Tarone–Ware tests analyses were conducted to compare the 5-year survival between various patient cohorts and *t*-tests, chi squared tests, and 1- and 2-way analyses of variance (ANOVA) were conducted to compare patient characteristics between groups.

## 3. Results

There were 122 patients under 50 who were diagnosed with OST seen at Rush Medical Center in the past 25 years who had a presenting CT scan on file. Twenty-five patients were excluded: 8 because their initial micronodule(s) were surgically removed, 14 because they had at least one nodule ≥10mm, and 3 because of a history of another malignancy. We had 2- and 5-year survival data on 85% and 72% of included patients, respectively. Our data showed an approximately equal distribution of nodules from 1–4 mm from 1995 to 2020, suggesting similar CT sensitivity throughout the study.

**Figure 1:**
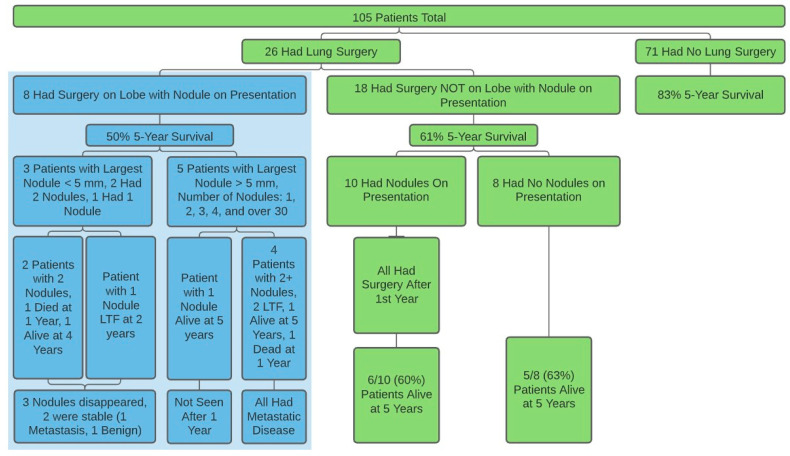
The number of patients who had surgery and the overall survival for each group. Patients represented in the blue box were excluded from the study because they all had surgery on the lobe with nodule involvement on presentation. LTF = Lost to follow up.

There were 97 patients (mean age = 20, range = 6–49, 46 F, 51 M) that fit inclusion criteria: 53 with no nodules and 44 with at least one nodule, with 45% “good responders” (Huvos 3 or 4). The 5-year survival for the 18 patients who had surgery or radiofrequency ablation involving nodules not involved on presentation, and the 79 patients who had no surgery at any time were 64% and 92%, respectively (Figure 1, Table S1). There were no differences found on size or number of nodules on presentation or within one year between demographic groups investigated.

**Figure 2:**
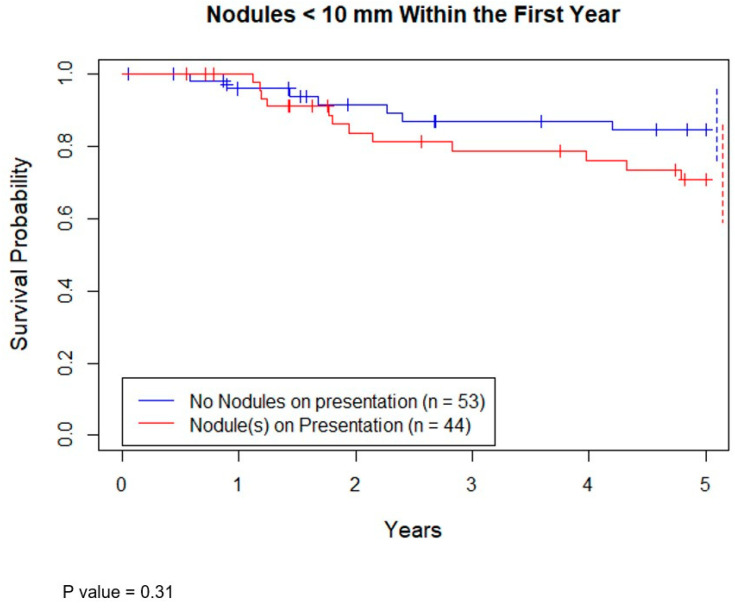
Kaplan–Meier curve demonstrating survival probability vs. years. Groups are separated by presence of a nodule < 10 mm on presentation and those who did not have any nodules on presentation. Dotted lines represent 95% confidence intervals for respective color.

Seventy-eight patients had survival data at 5 years. Of these, 61 were still alive (78%). The 5-year overall survival (OS) of patients with at least 1 nodule < 10 mm on presentation versus none showed no statistical difference (Figure 2, *p* = 0.31).

### 3.1. Survival Based on Size of Largest Micronodule

**Figure 3:**
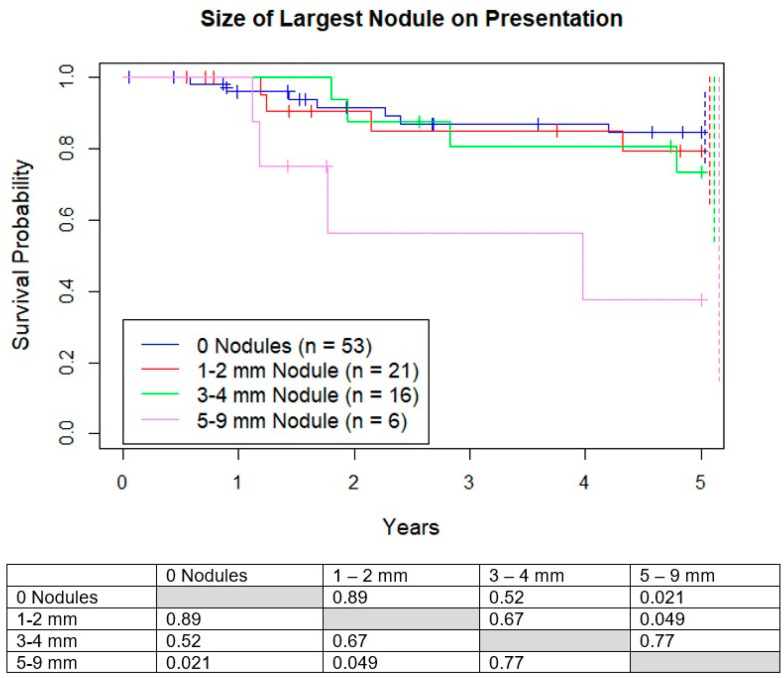
Kaplan–Meier curves demonstrating survival probability vs. years. Groups are separated by size of largest micronodule on presentation. Dotted lines represent 95% confidence intervals for respective color.

We analyzed four subgroups based on the size of the largest nodule: no nodules, 1–2 mm, 3–4 mm, and 5–9 mm. Five-year OS was 83%, 82%, 73%, and 40% respectively, with much lower survival for the 5–9 mm group compared to the other 3 groups (Figure 3). There was no difference in 5-year survival between the groups <5 mm. There was no difference in percentage of good responders between the 4 groups (*p* = 0.91).

### 3.2. Survival Based on Number of Micronodules

**Figure 4:**
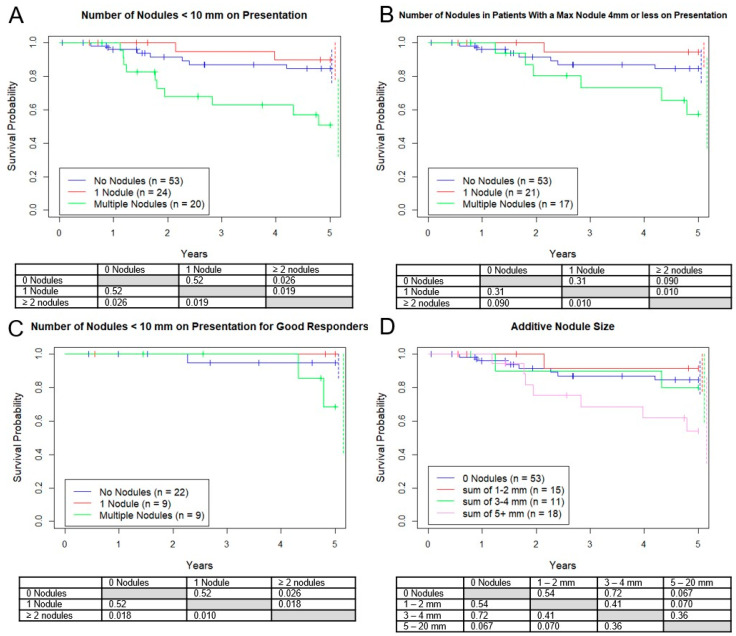
Kaplan–Meier curves demonstrating survival probability vs. years. Groups separated by (**A**) number of nodules, (**B**) number of nodules <5 mm, (**C**) number of nodules <10 mm restricted to “good responders”, or (**D**) sum of diameter. Dotted lines represent 95% confidence intervals for respective color.

Additionally, we analyzed three subgroups based on the number of presentation nodules. The OS for no nodules was 83%, versus 90% for 1 nodule, and 53% for 2 or more (Figure 4A). However, when only including patients with nodules all <5 mm (Figure 4B) or the good responders (Figure 4C) this difference was not seen. All 3 groups had approximately the same proportion of good responders (42%, 43%, 55%, *p* = 0.61).

Similar to the RECIST v. 1.1 paradigm, estimates of disease burden for “target” nodules >10mm, we analyzed four subgroups based on the sum of the diameters of all micronodules. Five-year survival rates for 0 mm total, 1–2 mm total, 3–4 mm total, and 5–20 mm total were 83%, 91%, 80%, 53%, respectively, with no statistical survival differences between any of the groups (Figure 4D). However, there was a trend towards a worse outcome for the group with a sum of 5–20 mm vs. 0–2 mm (*p* = 0.070). As before, there was no significant difference in good responders between these groups.

### 3.3. Survival Based on Number of Lobes Involved

**Figure 5:**
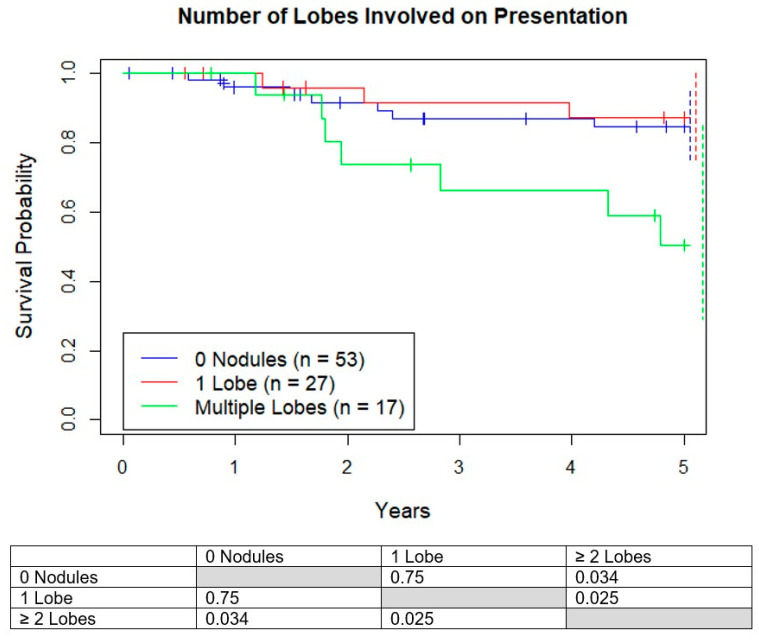
Kaplan–Meier curves demonstrating survival probability vs. years. Groups are separated by number of lobes with lung nodules < 10 mm on presentation. Dotted lines represent 95% confidence intervals for respective color.

Next, the number of involved lobes on presentation were analyzed. Survival for those who presented with 0 lobes, 1 lobe, and multiple lobes involved were 83%, 87%, and 50%, respectively, with 2 or more involved lobes having worse life expectancies (Figure 5). The average size of the nodules in patients with one versus multiple involved lobes were similar (2.6 vs. 3.4 mm, *p* = 0.10) and the average number of nodules (1.1 vs. 3.1, *p* = 0.00049) were significantly different.

### 3.4. Survival with New Micronodules after Diagnosis within 1 Year

**Figure 6:**
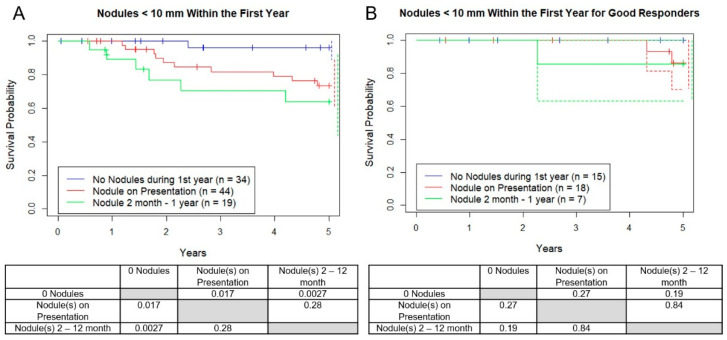
Kaplan–Meier curves demonstrating survival probability vs. years. Groups are separated by number of micronodules at diagnosis vs. those who developed nodules after initiation of systemic therapy within one year of diagnosis (2–12 months from diagnosis) for (**A**) all patients and (**B**) patients categorized as “good responders.” Dotted lines represent 95% confidence intervals for respective color.

Next, the development of new nodules by the first year after diagnosis were analyzed. Nineteen of the 53 patients (36%) who originally showed no micronodules developed them in the first year (mean age = 21, 11 F, 8 M), and of those, 12 while on chemotherapy (23%) and 7 after chemo (13%). Of the 12 patients who developed their first nodules on chemotherapy that we have confirmed status after 5 years, 3 out of 9 (33%) survived and all 7 out of 7 patients who developed their first nodules after chemotherapy survived at least 5 years. OS between patients in the “never developed nodules in the first year” versus “any micronodules at presentation” versus “new micronodule after presentation” was worse for each group respectively (Figure 6A, 96%, 73%, 63%, *p* = 0.017, 0.002). There was no difference between good responders among these three groups (*p* > 0.73). As before, when only good responders were included this difference disappeared (Figure 6B).

## 4. Discussion

The 5-year all-cause survival rate of patients with OST ranges from 28% to 70% [1,2]. This variation has been attributed to metastasis at diagnosis, age, tumor location, tumor size, and percent necrosis at primary surgery. However, it has not been well established how the discovery of lung micronodules at the time of diagnosis effects the 5-year overall survival and management. Our retrospective, single institution study of 97 patients demonstrated a 78% all 5-year survival rate and a “good responder” rate of 45%, which are in agreement with historical “localized” OST 5-year survival rates [12,19,20] suggesting our patient population was representative.

In our study, there was no difference in 5-year OS for those who presented with an additive nodule size <5 mm, or a solitary nodule <10 mm, or any number of nodules <5 mm, compared to having no presenting nodules (78–83%), similar to historical survival rates for localized disease (65–70%) [4,21], while the patients with 5–9 mm presenting nodules, though too small to draw conclusions, had a worse 5-year OS (40%) similar to historical survival rates for “lung only” metastatic disease (55%) [22]. Not surprisingly, patients with new micronodules after presentation and while still on systemic therapy had a significantly lower 5-year OS (63%). This difference vanished when only good responders were considered, suggesting this metric remains a more important feature. However, these comparisons involved a relatively small number of patients and, therefore, further investigations are needed to determine the role Huvos scores plays in lung nodule management. Of all our findings, the number of nodules is our best supported finding and therefore may be the most important clinically.

Previous studies have found that up to 25% of patients have metastatic lung nodules on presentation [23,24]. Our study identified 45% of patients to have presentation micronodules, similar to Ghosh et al. in 2018 (47%) [18], since we included all patients with micronodules not explicitly identified as benign on CT, which ultimately includes metastatic disease in addition to AVMs, atelectasis, and infectious causes like histoplasmosis. Regardless, Kusma et al. argue that nodules <5 mm in OST warrant biopsy/resection because of this high incidence of malignancy found upon histology (63%) [17]. However, our question was not if the micronodules are malignant, but if it is relevant to survival. It is plausible that a patients’ immune system and chemotherapy are adequate to prevent the progression of these micronodules as Link et al. established in 1986 [25], before the availability of thin-slice CT scanning, with “negative” lung CT scans. The sufficiently small lung nodule that we are now able to “see” likely represents the lung “micrometastasis” we have always known to exist in the majority of OST patients.

Harting et al. previously found a benefit to aggressive surgical resection of all resectable pulmonary nodules reporting a 5-year survival advantage (33.6 months vs. 10.1 months) for patients who undergo thoracic “clean out” surgery regardless of tumor characteristics. However, their study looked at a different patient population. In their study, the majority (83%) of nodules were found after presentation, i.e., while on systemic therapy, all nodule sizes were included, and the non-resection control group involved patients with unresectable disease (42%) and death before surgery (16%) [26].

Surgical intervention is not without side effects. Besides the delay in definitive treatment and anxiety for patients and families, intraoperative and postoperative complications in OST lung surgery occur 12% and 8% of the time, respectively [26]. As imaging continues to increase sensitivity, understanding the relevance of possibly clinically insignificant subcentimeter nodules is critical to avoid excessive treatment.

There are a limitations that need to be addressed. The study is single institution (which also provided uniformity of treatment, radiology, and surgery) and was retrospective. The total number of patients, 97, could have been increased if we included patients over 50; however, we felt that this would confound our analysis with a higher incidence of lung cancer, infectious granulomas, differing treatment paradigms, and more comorbidity. There were generally fewer patients in the ‘larger size’ and ‘number of nodules’ groups, and conclusions would be strengthened, or even changed, if patient numbers in these groups were increased. Additionally, we relied on the CT radiology report to determine the presence and size of nodules, and there may be some inconsistencies among radiologists. However, in practice, community oncologists determine treatments based on these radiology reports and we hope to provide practical information for them. We hope that prospective randomized studies that include observation versus biopsy of micronodules, especially in the subset of “good responders,” can be performed to help answer these questions.

## 5. Conclusions

At our institution, micronodules are found in approximately half of newly diagnosed OST younger than 50. Our data show 5-year overall survival for patients with micronodule(s) at presentation, especially if the patient is a good responder to chemotherapy, behave like historical “localized” OST. While for a subset, particularly poor responders, who have 2 or more nodules, have nodules ≥ 5 mm, or development of new nodules in the first year while on systemic chemotherapy, behave intermediate to historical localized and metastatic patients. As imaging continues to increase in sensitivity, understanding the relevance of sub-centimeter nodules is critical to properly managing, counseling, and classifying OST patients with pulmonary micronodules. Taken together, our data suggest that timing of nodule appearance, nodule size, number, and if the patient is a good responder, are key components to developing criteria on which nodules should and should not require surgical intervention.

## Data Availability

The data presented in this study are available on request from the corresponding author. The data are not publicly available due to patient confidentiality and local IRB rules.

## Supplementary Materials

The following are available online at https://www.mdpi.com/2077-0383/10/6/1213/s1, Table S1: Nodule Data on 8 Patients with Surgical Resection of Nodules on Presentation.
