# Supplementary material for: What Effect Do Pulmonary Micronodules Detected at Presentation in Patients with Osteosarcoma Have on 5-Year Overall Survival?
Source: J Clin Med. 2021 Mar 15;10(6):1213. doi: 10.3390/jcm10061213 (PMC8002003; doi:10.3390/jcm10061213)
Supplement: Supplementary file 1 [file jcm-10-01213-s001.pdf]

Table S1: Nodule Data on 8 Patients with Surgical Resection of Nodules on Presentation

	CT On Presentation		CT Prior to First Lung Surgical Intervention				Status	Comments
Patient	Number of Nodules	Size (mm)	Time After Presentation	Number of Nodules	Size (mm)	Percentage Malignant		
1	3	8, 5, 1	Immediately	3	8, 5, 1	66%, (1 mm nodule had "no residual tumor cells")	Lost to follow up at 1 year	
2	2	2, 2	1 year	4	3, 2, 2, 2	100%	Diseased at 1 year	Resected because of 3 new nodules. One of original nodules stable at 2 mm, the other was not visible on second CT
3	2	2, 2	1.5 years	7	7, 3, 2x5	100% Only 7 mm nodule was removed	Alive at 4 years	Original 2 nodules not removed during first procedure, 1 disappeared after 1 year, the other was removed 2 years later and was benign
4	1	2	2 years	1	8	100%	Lost to Follow up at 2 years	Original nodule disappeared within 1 year
5	4	6, 2x3	0.5 years	4	8, 3, 2x2	75%	Alive past 5 years	2 of 4 original nodules grew in size and were both metastases
6	1	7	2 years	1	10	100%	Alive past 5 years	Surgery did not involve original nodule, Original nodule remained stable
7	2	8, 1	3 months	Many	Many over 10	100%	Diseased at 1 year	Original 8 mm nodule grew to 12 mm, 1 mm nodule remained stable and was not removed
8	32*	9, 8, 1x30	1 year	Many	Many over 10	Majority	Lost to Follow up at 3 years	Original 9 and 8 mm nodules grew to 11 and 9 mm, respectively, both metastases

* Indicates an estimation of number of nodules
